# National estimates for maternal mortality: an analysis based on the WHO systematic review of maternal mortality and morbidity

**DOI:** 10.1186/1471-2458-5-131

**Published:** 2005-12-12

**Authors:** Ana P Betrán, Daniel Wojdyla, Samuel F Posner, A Metin Gülmezoglu

**Affiliations:** 1 UNDP/UNFPA/WHO/World Bank Special Programme of Research, Development and Research Training in Human Reproduction, Department of Reproductive Health and Research, World Health Organization, Geneva, Switzerland; 2 Centro Rosarino de Estudios Perinatales, and Escuela de Estadística, Universidad Nacional de Rosario, Rosario, Argentina; 3 Division of Reproductive Health, Centers for Disease Control and Prevention, Atlanta, GA, USA

## Abstract

**Background:**

Despite the worldwide commitment to improving maternal health, measuring, monitoring and comparing maternal mortality estimates remain a challenge. Due to lack of data, international agencies have to rely on mathematical models to assess its global burden. In order to assist in mapping the burden of reproductive ill-health, we conducted a systematic review of incidence/prevalence of maternal mortality and morbidity.

**Methods:**

We followed the standard methodology for systematic reviews. This manuscript presents nationally representative estimates of maternal mortality derived from the systematic review. Using regression models, relationships between study-specific and country-specific variables with the maternal mortality estimates are explored in order to assist further modelling to predict maternal mortality.

**Results:**

Maternal mortality estimates included 141 countries and represent 78.1% of the live births worldwide. As expected, large variability between countries, and within regions and subregions, is identified. Analysis of variability according to study characteristics did not yield useful results given the high correlation with each other, with development status and region. A regression model including selected country-specific variables was able to explain 90% of the variability of the maternal mortality estimates. Among all country-specific variables selected for the analysis, three had the strongest relationships with maternal mortality: proportion of deliveries assisted by a skilled birth attendant, infant mortality rate and health expenditure per capita.

**Conclusion:**

With the exception of developed countries, variability of national maternal mortality estimates is large even within subregions. It seems more appropriate to study such variation through differentials in other national and subnational characteristics. Other than region, study of country-specific variables suggests infant mortality rate, skilled birth attendant at delivery and health expenditure per capita are key variables to predict maternal mortality at national level.

## Background

Since the launching of the Safe Motherhood Initiative in 1987 [1], there has been a worldwide effort to reduce maternal mortality and to identify its determinants. These efforts have been directed by the outputs of a number of international conferences over the past decade such as the International Conference on Population and Development in 1994, and the Fourth World Conference on Women in 1995 reinforced this commitment. The declaration of the Millennium Development Goals (MDGs) aiming at reducing by three-quarters the maternal mortality ratio between 1990 and 2015 has also increased the demand for measuring maternal mortality at national and subnational levels [2].

Despite worldwide concern, an outstanding problem is how to monitor maternal mortality and to obtain reliable and comparable data. Measuring maternal mortality accurately is notoriously difficult except where there is comprehensive registration of deaths and causes of death. Unfortunately, there are only a few countries where such registration could be characterized as complete [3] and even in these countries, poor attribution of cause of death results in significant underreporting of maternal deaths [4,5]. In addition, countries with complete death registration are countries with low maternal mortality, and, consequently, countries where it is not a public health priority. It is in countries where a reliable vital registration system is not in place where maternal mortality represents a public health problem that cannot be accurately measured.

Several alternative techniques have been developed to fill the gap caused by poorly functioning vital registration systems. Of these, the Reproductive Age Mortality Studies (RAMOS) are considered the gold standard for measuring maternal mortality because it involves identifying and investigating the causes of all deaths of women in reproductive age [6]. Another approach currently used in most developing countries derives estimates of maternal mortality from household surveys or surveys using the sisterhood method [7]. The sisterhood method is an indirect measurement technique that reduces sample size of the surveys by interviewing respondents about the survival of all their sisters [7]. Data on maternal deaths obtained through census has also been proposed as a means of estimating levels of maternal mortality [8]. Drawbacks include high costs in the case of RAMOS, large sample sizes required for household surveys and the use of estimates intrinsically referring to the past instead of the current situation in the case of sisterhood methods. Differentials in the definition of maternal death, varying efforts carried to capture maternal deaths, and the methods used to confirm the deaths as 'maternal' are some of the inherent discrepancies in these methods that may affect estimates and impede comparisons. Unfortunately, a measure allowing for comparisons between these methods is lacking.

WHO, jointly with UNICEF and UNFPA, has made efforts to monitor maternal mortality by producing global, regional and national estimates for 1990, 1995 and 2000 [3,9,10]. Different methodologies used to calculate maternal mortality ratios as well as the lack of national data for many of the countries have been identified as major problems in assessing the global situation as well as for monitoring trends. Estimates for 2000 suggested 529,000 maternal deaths worldwide with an average maternal mortality ratio of 400 per 100,000 live births, and accounted for 173 countries with 99% of global births. However, 62 countries (27% of global live births) had no national data available, and maternal mortality estimates for those countries were developed using a regression model based on a set of explanatory country-specific variables that are available for nearly all countries in the world [3]. An alternative model based also on country-specific variables was also proposed using the same data set [11].

The UNDP/UNFPA/WHO/World Bank Special Programme of Research, Development and Research Training in Human Reproduction (HRP), Department of Reproductive Health and Research at the World Health Organization has conducted a systematic review of the prevalence/incidence of maternal mortality and morbidities from 1997 to 2002 worldwide. The primary objective of this review is to assist in mapping the burden of reproductive ill-health by providing a comprehensive, standardized and reliable tabulation of data on the incidence/prevalence of maternal morbidity and mortality [12]. This article presents an analysis of the nationally representative maternal mortality data included in the review exploring the correlation between the maternal mortality estimates with study-specific and country-specific variables.

## Methods

The methodology of the systematic review has been described elsewhere [13]. In brief, we searched for published and unpublished data on maternal mortality and morbidity reported between 1997 and 2002. The search strategy included 10 relevant electronic databases, hand searching, screening of reference lists of retrieved articles, congress abstract books, and contacting experts active in the field. Furthermore, we searched databases in developing countries such as Index Medicus of the Eastern Mediterranean Region (IMEMR) [14], African Index Medicus (AIM) [15]; IndMED [16], a bibliographic database of Indian biomedical journals; and HELLIS.ORG [17], a network of health science libraries across Asia. Results on the effectiveness of searching the different databases have been published elsewhere [18].

**Figure 1:**
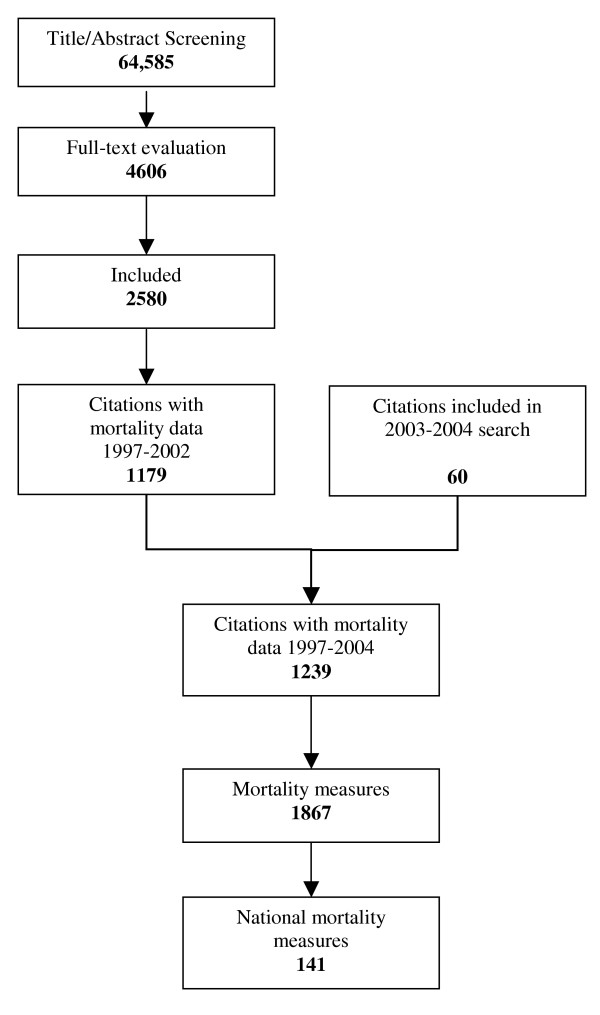
**Citations flow chart of the systematic review**. Flow chart of citations and maternal mortality measures included in the systematic review. Note that one citation could provide several maternal mortality measures.

Criteria for inclusion of studies in the review were: inclusion of data on prevalence/incidence of maternal mortality or morbidity, specified dates for data collection period, including data from 1990 onwards, sample size larger than 200 and a clear description of methodology using pre-defined criteria. In order to bring these estimates up to date, during 2003 and 2004 we performed an active search for data on mortality at national level and included the records identified. Figure 1 shows the flow diagram of the process.

We extracted data from included studies using a specifically designed data extraction form which included 48 questions distributed in five modules. The three modules related to levels of maternal mortality were designed to collect information on: (i) general characteristics of the study such as design, population and setting, (ii) maternal mortality measure, (iii) characteristics of the mortality measure which included definition of maternal death if reported, and whether or not efforts were made to capture all maternal deaths and to confirm them [13]. Although there are three distinct measures in use expressing levels of maternal mortality – ratio, rate and lifetime risk of maternal death – the most commonly used measure is the maternal mortality ratio (MMR), that is, the number of maternal deaths during a given period of time per 100,000 live births during the same period of time. The MMR is the measure used in this analysis.

### Process to select nationally representative estimates

This manuscript presents results based only on the nationally representative data identified through the systematic review. For 32 countries more than one estimate was available from different study designs. In these cases a judgement was made concerning the most recent and the best estimate (e.g. direct sisterhood would be selected over indirect sisterhood method and RAMOS over vital registration if both are available for the same year). When data were available for several years using the same study design (the case of developed countries with data from vital registration systems), the most recent was considered except when the number of deaths reported was less or equal to 10 in which case an average of the last three years was considered. If several definitions of maternal mortality were available, that of the International Classification of Diseases (ICD-10), specifying deaths up to 42 days postpartum was considered [19]. If the same data set was presented in multiple reports, that providing the most information was considered.

Coverage of available estimates at regional or subregional level is given as a percentage of the total number of live births in the region or subregion. Estimates of the number of live births, and the regional, subregional and development status classifications used, are based on those of the United Nations (UN) [20].

### Statistical analysis

To analyze maternal mortality estimates we grouped countries according to UN region (Africa, Asia, Europe, Latin America and the Caribbean, Northern America and Oceania), subregion and country development status (developed, less developed, least developed). For both regional and subregional analyses, Europe, USA, Canada, Japan, Australia and New Zealand were grouped together. Boxplots are used to display variability in national estimates [21]. We included a measure of coverage by calculating the proportion of live births within the region/subregion covered by the maternal mortality estimates in the analysis. We explored the relationship between study-specific and country-specific variables with MMR. Study-level variables were: study design, sampling method, source of data, information regarding the proportion of non-responders or completeness of records, definition of maternal death, confirmation of maternal death, and type of effort used to ensure capture of all maternal deaths. "Study design" refers to the type of design or method from which the estimate was derived and is not meant to imply that an actual study was conducted. Designs included here are census, vital registration, direct sisterhood, indirect sisterhood, direct survey, RAMOS and confidential enquiries into maternal deaths (CEMD) (See Table 1).

**Table 1:** Characteristics of studies. Characteristics of the studies and reports deriving national MMR estimates by region.

Variables	Africa	Asia	Latin America & Caribbean	Developed Regions	Total
**TOTAL**	**35**	**36**	**27**	**43**	**141**

**Study design/method**					
Census	0	1	0	0	1
Cross sectional (Vital registration)	3	18	16	42	79
Direct sisterhood method	22	7	5	0	34
Indirect sisterhood method	6	2	3	0	11
Direct survey	1	2	0	0	3
RAMOS^a^	3	5	2	0	10
CEMD^b^	0	1	1	1	3
					
**Sampling**					
Random	29	11	8	0	48
All women	6	25	19	43	93
					
**Data reported**					
Estimates	28	12	8	1	49
Actual counts	6	23	18	42	89
Mixed/Other	1	1	1	0	3
					
**Information on lost to follow-up/non-respondents**					
Yes	29	12	8	1	50
No	6	24	19	42	91
					
**Maternal death definition**					
ICD9=ICD10 up to 42 days pp	4	23	15	41	83
ICD10 up to 60 days pp.	1	0	1	0	2
ICD10 up to 1 year pp.	1	0	0	1	2
Preg-related up to 42 days pp.	5	2	2	0	9
Preg-related up to 60 days pp.	22	6	5	0	33
No Maternal Death Definition	2	5	4	1	12
					
**Maternal deaths confirmed**					
Confidential enquiry	1	0	3	1	5
Verbal autopsy	1	4	1	0	6
Confidential enquiry & verbal aut.	3	1	1	0	5
Multiple sources	0	1	0	0	1
Unknown	1	1	0	0	2
Not applicable^c^	27	8	7	0	42
No confirmation maternal deaths	2	21	15	42	80
					
**Special efforts to capture all maternal deaths**					
Multiple data sources	2	3	3	2	10
Identifying deaths of WRA^d^	2	2	0	0	4
Special enquiry/interview	29	10	8	0	47
Yes but unspecified	0	0	1	0	1
No	2	21	15	41	79

^a^RAMOS: Reproductive Age Mortality Studies

^b^CEMD: Confidential Enquiries into Maternal Deaths

^c^Includes studies using a pregnancy-related death definition for which ascertainment of cause of death does not apply.

^d^WRA: Women in reproductive age.

A selected number of country-specific demographic and health variables were considered to explore their association with MMRs. These variables represent a broad array of factors that may influence maternal mortality and include population growth rate, probability of death between 15 to 59 (male), proportion of urban population, infant mortality rate, proportion of births attended by skilled health professional (referred in this manuscript as skilled birth attendant), contraceptive-use prevalence rate, health expenditure per capita and female net primary school enrolment ratio. Data for these variables were obtained from the Population Division of the United Nations [20,22,23] except for skilled birth attendant, health expenditure per capita, and probability of death between 15 to 59 (male) which were obtained from WHO databases [24,25]. Both standard analysis of variance and a non-parametric analogue comparing medians (Kruskal-Wallis) were used to identify differences between regions in country-level variables.

A regression analysis was used to identify independent associations between the country-specific variables selected from the previous analysis of variance and MMR. Initially, all variables identified in the analysis of variance along with region indicators were included in a regression model where MMR was considered as response variable. Variables were dropped when they were not statistically significant or when they caused co-linearity problems, using a variance inflation value of eight as a cut-off point to exclude variables co-linear with others regressors in the model. In both the analysis of variance and regression analysis, MMR and health expenditure per capita were log transformed because the distributions were highly skewed. Analysis was performed using the Statistical Analysis Software (SAS, Cary, North Carolina).

## Results

### Descriptive analysis of data set

Nationally representative estimates of maternal mortality are available for 141 countries representing 78% of the world live births. Additional file 1 presents the list of countries for which national MMR were identified through the systematic review, and, for each country, shows the ratio, period of time to which the estimate refers and the study design. For the majority of countries only one estimate based on one type of study design was available. However, for 32 countries national estimates derived from different study designs were available. For these, we took nine RAMOS over any other study design; 15 direct sisterhood over indirect sisterhood method, older censuses or vital registration data (all in developing countries); three direct surveys over direct and indirect sisterhood method or vital registration data; two vital registration data over older census (all in developed countries); and three indirect sisterhood over older direct sisterhood method or vital registration data.

Table 1 shows the regional distribution of selected study characteristics. More than half of the country estimates (56%) were derived from vital registration, followed by surveys including those using sisterhood methods (34%). Ten countries presented maternal mortality estimates derived from RAMOS studies. In 12 countries, reports did not provide a definition of maternal death, while 60% used the definition of the ICD-10 (i.e. a definition based on cause of death) up to 42 days postpartum. About 30% used a pregnancy-related definition (i.e. a definition based on time of death). More than half of the national estimates (57%) did not use any method to confirm maternal deaths. The same proportion did not report the use of special efforts to capture maternal deaths.

### Analysis of study-specific variables

**Figure 2:**
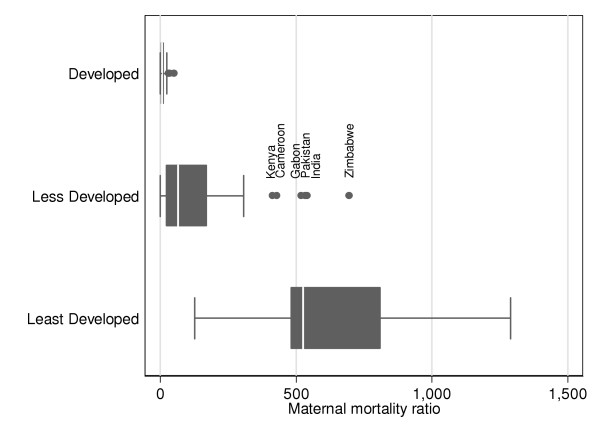
**National MMRs and development status**. Boxplot for national MMRs classified according to development status.

There is substantial variability in maternal mortality estimates between countries even when grouped according to development status or region (with the exception of more developed countries). Figure 2 shows boxplots comparing maternal mortality estimates by development status. The median is shown by the line inside the box, the box includes the inter-quartile range and the fences indicate 1.5 times the inter-quartile range. Outliers are shown by dots and correspond to values beyond 1.5 times the inter-quartile range. Outliers for less developed countries are Zimbabwe (MMR = 695 per 100,000 live births), India (540), Pakistan (533), Gabon (519), Cameroon (430) and Kenya (414). All these countries present estimates derived from sisterhood method, and have wide confidence intervals.

**Figure 3:**
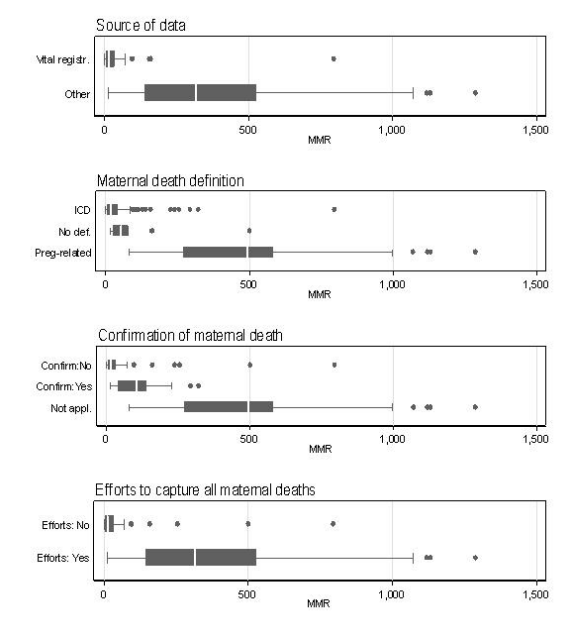
**National MMRs and study-specific variables**. Boxplots for national MMRs classified according to selected study-specific variables. In vertical order: (1) Source of data: vital registration versus others sources including surveys, Reproductive Age Mortality Studies (RAMOS) and Confidential Enquiries into Maternal Deaths (CEMD); (2) Definition of maternal death: ICD definition, pregnancy-related definition or no definition available; (3) Confirmation of maternal deaths: yes, no or not applicable; (4) Efforts to capture all deaths (yes or no).

Study design dictates other study-specific variables (e.g. sampling method, source of data, and definition of maternal death) and it is highly correlated with development status and region. In other words, once one of these variables has been fixed the others are mostly determined. To illustrate this point, the boxplots presented in Figure 3 show the variation in MMR according to selected study-specific variables. For example, in general, estimates from developed countries are derived from vital registration, use the ICD definition of maternal death, maternal deaths are not confirmed and no efforts to capture all deaths are made. On the other hand estimates from developing countries are derived from other sources (mostly surveys, with several RAMOS) which use a pregnancy-related definition due to limitations on data collection and therefore confirmation of maternal death does not apply because all deaths, maternal and not maternal, are included.

To further illustrate this high correlation, we grouped the 141 national MMRs according to four major study-specific variables: study design, source of data, maternal death definition and confirmation of maternal death. Some 121 (86%) of national estimates are captured by two specific combinations of these four variables. Seventy-nine estimates derive from cross-sectional designs using vital statistics with a definition based on the ICD or no definition reported and with no confirmation of maternal deaths. Forty-two estimates derive from surveys (source of data interviews) using a pregnancy-related maternal death definition, and therefore, confirmation of maternal death is not applicable.

**Figure 4:**
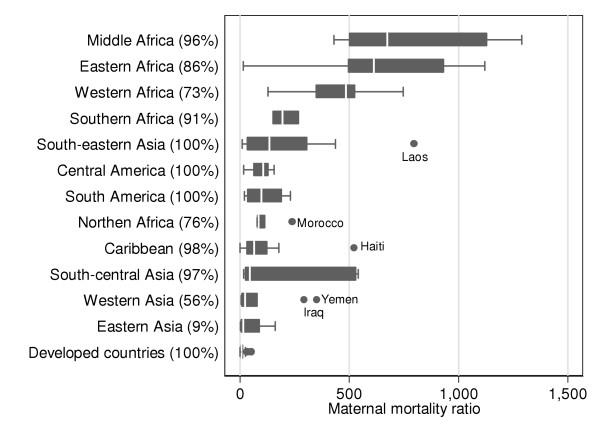
**National MMRs and subregion**. Boxplot for national MMRs classified according to UN subregion (developed countries are all grouped together). Japan is excluded from Eastern Asia and included in developed countries. Developed countries included therefore: Europe, USA, Canada, Japan, Australia and New Zealand. Oceania is not shown since data are only available for Australia and New Zealand which are shown under developed countries. Percentages shown next to the name of each subregion are the coverage, in terms of live births, achieved by available estimates).

Figure 4 shows levels and variability of MMRs by subregion (developed countries are shown together). Coverage (in terms of live births, shown next to the name of the subregion) ranges from 100% in developed regions, Central and South America, and South-eastern Asia to 9% in Eastern Asia due to the lack of a national-level maternal mortality estimate for China. All subregions except for two have coverage levels higher than 70%. As shown in Figure 4, four of the five African subregions have the highest MMR. Within these subregions there are substantial differences in variability, with Middle and Eastern Africa showing the largest inter-country variability. In addition, both South-eastern and South-central Asia show substantial variability in national estimates. Outliers at subregional level are, for South-eastern Asia (Laos, MMR = 796), Northern Africa (Morocco, MMR = 238), Caribbean (Haiti, MMR = 523), and Western Asia (Yemen, MMR = 351; Iraq, MMR = 294).

### Analysis of country-specific variables

Table 2 presents the medians and inter-quartile ranges by region (developed countries grouped together) for country-specific variables considered potentially useful for predicting maternal mortality. Standard analysis of variance and non-parametric ANOVA provided similar results. Non-parametric ANOVA results were used to identify variables with significant median differences across regions. The goal of this analysis was to identify measures that could be excluded from the regression analysis due to a lack of variability across regions. Analysis was conducted by region rather than subregion to maximize statistical power. Significant inter-regional differences were found for all variables selected for inclusion in the analysis.

**Table 2:** Summary statistics used in the model. Summary statistics by region. For each country-specific variable in the model, the number of MMR estimates, the median and the interquartile range by region are shown*.

Variable	**Africa**		**Asia**		**Latin America & Caribbean**		**Developed Countries**	
	
	N	Median (1^st ^– 3^rd ^Q)	N	Median (1^st ^– 3^rd ^Q)	N	Median (1^st ^– 3^rd ^Q)	N	Median (1^st ^– 3^rd ^Q)
Maternal Mortality Ratio	35	498 (238 – 729)	36	37 (18 – 275)	27	71 (41 – 161)	43	7 (4 – 14)

Skilled birth attendant (%)	34	47.1 (39.0 – 61.8)	34	95.1 (56.4 – 98.4)	27	87.6 (65.0 – 98.2)	43	98.0 (98.0 – 99.1)
Infant mortality rate	35	86 (62 – 110)	35	37.0 (16 – 72)	27	30 (18 – 41)	43	7 (5 – 12)
Health expenditure per capita	35	47 (32 – 163)	34	169 (85 – 390)	26	352 (193 – 533)	43	1512 (454 – 2358)
Population growth rate	35	2.5 (2.0 – 2.8)	35	2.0 (1.2 – 2.6)	27	1.7 (0.9 – 2.1)	43	0.2 (-0.2 – 0.6)
Probability of death between 15 and 59 (male, per 1000)	35	402 (262 – 482)	34	143 (98 – 208)	26	128 (97 – 150)	43	67 (59 – 97)
Female net primary school enrolment ratio	35	69.0 (45.0 – 94.0)	32	90.0 (75.5 – 93.5)	26	92.5 (88.0 – 100.0)	42	97.0 (92.0 – 100.0)
Urban population (%)	35	33.4 (27.5 – 48.9)	35	55.8 (27.6 – 78.7)	27	65.4 (56.1 – 75.3)	43	68.5 (59.4 – 83.3)
Contraceptive-use prevalence rate	35	26 (12 – 40)	33	55 (39 – 66)	23	62 (53 – 69)	35	74 (58 – 77)

* Difference among regions for all variables was statistically significant (p < 0.001), Kruskal-Wallis.

Initially, a full model including all country-specific variables along with a categorical predictor for country region was adjusted. Five of the eight variables were excluded due to lack of statistical significance or co-linearity problems (i.e. population growth rate, probability of death between 15 and 49 -male-, female net primary school enrolment ratio, proportion of urban population, and contraceptive-use prevalence rate). To be sure that none of the excluded variables were individually significant, a second set of models was fitted adding each of the five excluded variables to the model one at a time, but none of these variables were statistically significant. Table 3 presents the parameter estimates and standard errors for the final regression model. Ultimately, country region, infant mortality rate, health expenditure per capita and skilled birth attendant were statistically significantly associated with MMR. From the fitted model it can be deduced that countries in non-developed areas tend to present higher maternal mortality. The positive relationship between infant and maternal mortality suggests that an increase in infant mortality is associated with an increase in maternal mortality. Also, the regression coefficients indicate that increases in skilled birth attendant and health expenditure per capita are associated with decreases in maternal mortality. This model explained 90% of the variance in the log of the MMR. Potential interactions terms between region and the other independent variables were assessed but none of them improved the fit of the model.

**Table 3:** Regression model. Parameter estimates and their standard errors for the regression model of logarithm of maternal mortality ratio on selected country-specific demographic and health indicators.

Variable	Parameter Estimate	Standard Error	p-value
Intercept	5.465	0.621	<.0001
Region: Africa	1.329	0.229	<.0001
Region: Asia	0.748	0.174	<.0001
Region: Latin America & Caribbean	1.382	0.163	<.0001
Skilled birth attendant	-0.016	0.004	<.0001
Infant mortality rate	0.013	0.004	0.0003
Health expenditure per capita (log)	-0.272	0.075	0.0004

## Discussion

Analysis of study-specific variables shows a very clear cut-off between the different study designs, primarily surveys and vital registration (see Figure 3). Most of the estimates from developing countries come from surveys, and the inherent methodology entails certain study characteristics that are consistently different from estimates derived from vital registration, the established method in developed countries (e.g. sampling method, information on non-respondents or completeness of records, definition of maternal death). For example, whereas vital registration systems tend to use a definition of maternal death on the basis of cause of death, surveys tend to use a 'time-of-death' definition which includes all deaths that occur during pregnancy, childbirth or within a determined period of time after delivery regardless of the cause. Examination of the effect of different definitions on the estimates invariably groups countries according to their development status again, indicating close to complete confounding in this data set between study characteristics and country-level determinants of maternal mortality. For this reason, the effect of development status on the estimates is difficult to disentangle from the independent effect that other variables (especially study-design related) may have, and further analysis to explain variability at this level is not possible with this data set.

Interestingly, within all subregions except developed countries (Europe, Northern America, Japan, Australia and New Zealand) there is substantial variability between nations indicating the need for careful attention to country-level variables in understanding the specific issues of maternal mortality. In this analysis, inter-country comparison possibilities are very limited due to serious methodological differences between estimates. However, outliers in Figure 2 and 4 deserve special attention. There are six outliers in the less developed group in Figure 2 and four of these countries have MMRs greater than the median of the least developed countries. Although these countries do not fulfil the criteria to be classified as least developed, their high MMRs are more comparable to countries with the lowest development status. In-depth study of other possible economic, health, demographic and social differences could provide some light as to how to reduce maternal mortality in those settings.

Similarly, we can consider outliers at subregional level in Figure 4. For example, in Western Asia, a subregion with little variation between countries, Yemen (MMR = 351) and Iraq (MMR = 294) are clear outliers. Further research on other indicators at national level shows that these two countries present the lowest rates in the region for skilled birth attendant (21.6% and 72,1%, respectively), the lowest contraceptive-use prevalence rates (20.8% and 13.7%, respectively) and the highest infant mortality rates (80 and 95 per 1000, respectively).

The analysis of country-specific variables that might influence maternal mortality provides the opportunity to examine key factors in understanding the variability in maternal mortality. From the large number of country-specific variables that may be associated with maternal mortality we selected a few that represented a broad array of factors. These included variables proximal to maternal health including skilled birth attendant and contraceptive-use prevalence rate; intermediate variables such as health expenditure per capita; and distal measures such as population growth rate, proportion of urban population, probability of death between 15 and 59 (male) and female net primary school enrolment ratio. We reviewed the association between variables to identify independent indicators of the different factors.

A small number of these variables explained a substantial proportion of the variance observed in MMRs. These variables were all health related: skilled birth attendant, health expenditure per capita and infant mortality rate, and the first two have already been reported as determinants of maternal mortality using a smaller data set from sub-Saharan African countries [26]. Contraceptive-use prevalence rate, an indicator commonly used to describe access to health care for women was not independently associated with MMR in the multi-variate model. Although these indicators are all associated with development status, development status was not included in the analysis because it measures a wide range of issues and is potentially difficult to interpret and translate into programmatic recommendations. The findings here are supportive of the potential positive impact of skilled attendant at birth to reduce maternal mortality. Independent of this, increased health expenditure is also an important indicator supporting that not only skilled care but also general health infrastructure has an impact on maternal mortality.

Except for skilled birth attendant, the variables proposed here to predict maternal mortality are different than those used in the WHO model in 2000 (i.e. general fertility rate, skilled birth attendant, gross domestic product per capita, a regional variable, and a measure of completeness of death registration) [3], suggesting that further work towards understanding the covariates of maternal mortality would be useful. While the two exercises are not directly comparable (different dataset and modelling a different variable), it is useful to highlight certain contrasting findings in the belief that different approaches to the problem may each individually shed light on data gaps and on strategies to address them.

There are many country-level variables not included in this analysis that may also explain variability in MMR. In our initial analysis we considered a large number of potential indicators covering a broad range of factors. Many of these variables were highly correlated (r > 0.70). As part of our analysis plan we attempted to identify a sub-set of variables that covered different areas that were not highly inter-correlated but also variables most plausibly related to maternal mortality and that could be incorporated into public health programmes. While other variables may also explain variability, it is likely that they would point to similar set of programmatic activities.

Although this analysis presents national estimates from a large proportion of the world's live births, the lack of national estimates from China is the primary factor limiting near complete coverage of global information on maternal mortality. Data included in this analysis represent the most recent available estimate for each country. However, in some cases, especially in developing countries, most recent information could date from the 1980s given the retrospective aspect of sisterhood method estimates (even though the reporting date is after 1997). Changes in maternal mortality that may have occurred since the time of the study could influence both the variability and the identified associations. Normally, changes in maternal mortality, especially at national level, are slow, thus this would not likely impact the findings presented here. Furthermore, sisterhood methods, which include the oldest information presented here, essentially estimate an average across the time period so impact of rapid changes would be minimal.

In addition, data used for these analyses are derived from a variety of sources and methodologies, each presenting different pitfalls, constraints, precision and degree of reliability in identifying maternal deaths. For this reason, we do not attempt to provide here a summary estimate for maternal mortality. Previous attempts have been made to adjust for discrepancies, incompleteness or under-reporting in order to obtain estimates that may reflect the real situation better [3]. The data used here, however, are taken as reported, without adjustment, because we think that underreporting of maternal deaths must be estimated carefully through specific surveys tailored for each country [4,5]. This implies that MMRs presented here are likely to be underestimates, especially when we take into account that more than half of the estimates did not involve any efforts to capture maternal deaths by, for example, searching multiple sources, and the same proportion did not attempt to confirm maternal deaths through, for example, confidential enquiries or verbal autopsy [27].

The results of this analysis of maternal mortality at national level show significant variation for developing regions (from 127 to 1289 in least developed and 2 to 695 in less developed). Although due to co-linearity, an in-depth exploration of study-specific variables could not be done to explain the variability, the distribution and outliers suggests clearly specific countries as priority targets. On the basis of the analysis of correlation of MMRs with other country-specific variables, it seems prudent that a model to predict maternal mortality at national level takes into account infant mortality rate, skilled birth attendant and health expenditure per capita, as well as possibly other variables.

## Conclusion

With the exception of developed countries, variability of country maternal mortality estimates is large even within subregions. It seems more appropriate to study such variation through differentials in other national and subnational characteristics. Other than region, study of country-specific variables suggests infant mortality rate, skilled birth attendant and health expenditure per capita are key variables to predict maternal mortality at national level.

## Competing interests

The author(s) declare that they have no competing interests.

## Authors' contributions

APB, DW, SFP and AMG developed the idea for the analysis. DW performed the analysis. All four authors contributed to the interpretation of the data and writing of the manuscript.

## Pre-publication history

The pre-publication history for this paper can be accessed here:



## Supplementary Material

Additional File 1 **National estimates listing**. List of countries for which national MMRs were identified through the systematic review, and for each country, the ratio, period of time to which the estimate refers and the study design. Click here for file
