# Supplementary material for: National estimates for maternal mortality: an analysis based on the WHO systematic review of maternal mortality and morbidity
Source: BMC Public Health. 2005 Dec 12;5:131. doi: 10.1186/1471-2458-5-131 (PMC1351170; doi:10.1186/1471-2458-5-131)
Supplement: Additional File 1 — National estimates listing. List of countries for which national MMRs were identified through the systematic review, and for each country, the ratio, period of time to which the estimate refers and the study design. [file 1471-2458-5-131-S1.doc]

Additional File 1: National Estimates

| **Country** | **MMR** | **Period** | **Study Design** |
| --- | --- | --- | --- |
| **Eastern Africa** | | | |
| Comoros | 517 | 88-00 | Indirect sisterhood method |
| Eritrea | 998 | 86-95 | Direct sisterhood method |
| Ethiopia | 871 | 94-00 | Direct sisterhood method |
| Kenya | 414 | 93-03 | Direct sisterhood method |
| Madagascar | 488 | 90-97 | Direct sisterhood method |
| Malawi | 1120 | 94-00 | Direct sisterhood method |
| Mauritius | 24 | 98-00 | Cross-sectional |
| Rwanda | 1071 | 95-00 | Direct sisterhood method |
| Uganda | 505 | 96-01 | Direct sisterhood method |
| United Republic of Tanzania | 529 | 87-96 | Direct sisterhood method |
| Zambia | 729 | 95-02 | Indirect sisterhood method |
| Zimbabwe | 695 | 95-99 | Direct sisterhood method |
| **Middle Africa** | | | |
| Angola | 502 | 94-98 | Cross-sectional |
| Cameroon | 430 | 89-98 | Direct sisterhood method |
| Central African Republic | 1132 | 89-95 | Direct sisterhood method |
| Chad | 827 | 91-97 | Direct sisterhood method |
| Democratic Republic of the Congo | 1289 | 89-01 | Indirect sisterhood method |
| Gabon | 519 | 94-00 | Direct sisterhood method |
| **Northern Africa** | | | |
| Algeria | 114 | 99-99 | RAMOS |
| Egypt | 84 | 00-00 | RAMOS |
| Libyan Arab Jamahiriya | 77 | 83-95 | Indirect sisterhood method |
| Morocco | 238 | 92-96 | Direct Survey |
| Tunisia | 80 | 00-00 | Cross-sectional |
| **Southern Africa** | | | |
| Namibia | 271 | 91-00 | Direct sisterhood method |
| South Africa | 150 | 92-98 | Direct sisterhood method |
| Swaziland | 194 | 87-94 | Indirect sisterhood method |
| **Western Africa** | | | |
| Benin | 498 | 90-96 | Direct sisterhood method |
| Burkina Faso | 484 | 94-98 | Direct sisterhood method |
| Cape Verde | 127 | 92-93 | RAMOS |
| Guinea | 528 | 92-99 | Direct sisterhood method |
| Guinea-Bissau | 349 | 90-00 | Indirect sisterhood method |
| Mali | 582 | 94-01 | Direct sisterhood method |
| Mauritania | 747 | 94-01 | Direct sisterhood method |
| Nigeria | 289 | 93-99 | Direct sisterhood method |
| Togo | 478 | 93-98 | Direct sisterhood method |

| **Country** | **MMR** | **Period** | **Design** |
| --- | --- | --- | --- |
| **Eastern Asia** | | | |
| Hong Kong* | 2 | 97-99 | Cross-sectional |
| Japan | 7 | 01-01 | Cross-sectional |
| Mongolia | 160 | 00-00 | Cross-sectional |
| Republic of Korea | 20 | 96-96 | RAMOS |
| Taiwan, Province of* | 9 | 98-98 | Cross-sectional |
| **South-Central Asia** | | | |
| Bangladesh | 322 | 98-01 | Direct Survey |
| India | 540 | 97-98 | Direct sisterhood method |
| Iran (Islamic Republic of) | 37 | 96-96 | RAMOS |
| Kazakhstan | 44 | 00-00 | Cross-sectional |
| Kyrgyzstan | 54 | 02-02 | Cross-sectional |
| Nepal | 539 | 90-96 | Direct sisterhood method |
| Pakistan | 533 | 90-91 | Indirect sisterhood method |
| Sri Lanka | 20 | 96-96 | Cross-sectional |
| Tajikistan | 25 | 99-99 | Cross-sectional |
| Turkmenistan | 16 | 98-98 | Cross-sectional |
| Uzbekistan | 35 | 00-00 | Cross-sectional |
| **South-Eastern Asia** | | | |
| Brunei Darussalam | 27 | 00-00 | Cross-sectional |
| Cambodia | 437 | 94-00 | Direct sisterhood method |
| Indonesia | 307 | 98-03 | Direct sisterhood method |
| Lao People's Democratic Republic | 796 | 95-95 | Census |
| Malaysia | 28 | 00-00 | CEMD |
| Myanmar | 255 | 98-99 | Direct Survey |
| Philippines | 172 | 91-97 | Direct sisterhood method |
| Singapore | 11 | 99-01 | Cross-sectional |
| Thailand | 36 | 98-98 | RAMOS |
| Viet Nam | 95 | 99-99 | Cross-sectional |
| **Western Asia** | | | |
| Armenia | 28 | 00-02 | Cross-sectional |
| Azerbaijan | 79 | 90-00 | Indirect sisterhood method |
| Bahrain | 17 | 99-01 | Cross-sectional |
| Georgia | 22 | 98-00 | Cross-sectional |
| Iraq | 294 | 89-99 | Direct sisterhood method |
| Israel | 8 | 97-99 | Cross-sectional |
| Jordan | 40 | 95-96 | RAMOS |
| Kuwait | 6 | 98-00 | Cross-sectional |
| Qatar | 3 | 99-01 | Cross-sectional |
| Saudi Arabia | 23 | 97-98 | RAMOS |
| Yemen | 351 | 88-97 | Direct sisterhood method |

*Not WHO member state, special administrative region of China

| **Country** | **MMR** | **Period** | **Design** |
| --- | --- | --- | --- |
| **Eastern Europe** | | | |
| Belarus | 21 | 00-00 | Cross-sectional |
| Bulgaria | 17 | 02-02 | Cross-sectional |
| Czech Republic | 5 | 99-01 | Cross-sectional |
| Hungary | 8 | 00-02 | Cross-sectional |
| Poland | 4 | 01-01 | Cross-sectional |
| Republic of Moldova | 31 | 02-02 | Cross-sectional |
| Romania | 34 | 01-01 | Cross-sectional |
| Russian Federation | 34 | 02-02 | Cross-sectional |
| Slovakia | 14 | 01-01 | Cross-sectional |
| Ukraine | 24 | 01-01 | Cross-sectional |
| **Northern Europe** | | | |
| Denmark | 6 | 97-99 | Cross-sectional |
| Estonia | 21 | 99-01 | Cross-sectional |
| Finland | 5 | 00-02 | Cross-sectional |
| Iceland | 0 | 93-95 | Cross-sectional |
| Ireland | 4 | 97-99 | Cross-sectional |
| Latvia | 18 | 00-02 | Cross-sectional |
| Lithuania | 14 | 00-02 | Cross-sectional |
| Norway | 6 | 99-01 | Cross-sectional |
| Sweden | 3 | 99-01 | Cross-sectional |
| United Kingdom | 13 | 00-02 | CEMD |
| **Southern Europe** | | | |
| Albania | 8 | 99-01 | Cross-sectional |
| Bosnia and Herzegovina | 16 | 90-91 | Cross-sectional |
| Croatia | 6 | 00-02 | Cross-sectional |
| Greece | 4 | 97-99 | Cross-sectional |
| Italy | 3 | 00-00 | Cross-sectional |
| Malta | 31 | 98-01 | Cross-sectional |
| Portugal | 5 | 98-00 | Cross-sectional |
| Serbia and Montenegro | 10 | 97-99 | Cross-sectional |
| Slovenia | 13 | 99-01 | Cross-sectional |
| Spain | 4 | 00-00 | Cross-sectional |
| TFYR of Macedonia | 8 | 98-00 | Cross-sectional |
| **Western Europe** | | | |
| Austria | 4 | 00-02 | Cross-sectional |
| Belgium | 8 | 95-97 | Cross-sectional |
| France | 7 | 99-99 | Cross-sectional |
| Germany | 4 | 01-01 | Cross-sectional |
| Luxembourg | 6 | 99-01 | Cross-sectional |
| Netherlands | 9 | 00-00 | Cross-sectional |
| Switzerland | 6 | 98-00 | Cross-sectional |

| **Country** | **MMR** | **Period** | **Design** |
| --- | --- | --- | --- |
| **Caribbean** | | | |
| Bahamas | 32 | 93-95 | Cross-sectional |
| Barbados | 28 | 93-95 | Cross-sectional |
| Cuba | 42 | 02-02 | Cross-sectional |
| Dominican Republic | 178 | 92-02 | Direct sisterhood method |
| Haiti | 523 | 94-00 | Direct sisterhood method |
| Jamaica | 64 | 00-00 | Cross-sectional |
| Puerto Rico* | 22 | 92-92 | Cross-sectional |
| Trinidad and Tobago | 57 | 95-97 | Cross-sectional |
| **Central America** | | | |
| Belize | 111 | 94-96 | RAMOS |
| Costa Rica | 16 | 98-98 | Cross-sectional |
| El Salvador | 120 | 88-98 | Indirect sisterhood method |
| Guatemala | 190 | 90-95 | Direct sisterhood method |
| Honduras | 108 | 97-97 | RAMOS |
| Mexico | 64 | 02-02 | Cross-sectional |
| Nicaragua | 156 | 98-98 | Cross-sectional |
| Panama | 61 | 99-99 | Cross-sectional |
| **Southern America** | | | |
| Argentina | 41 | 99-99 | Cross-sectional |
| Bolivia | 230 | 97-03 | Direct sisterhood method |
| Brazil | 161 | 83-96 | Direct sisterhood method |
| Chile | 20 | 98-98 | Cross-sectional |
| Colombia | 35 | 94-94 | Cross-sectional |
| Ecuador | 97 | 01-01 | Cross-sectional |
| Paraguay | 192 | 89-95 | Indirect sisterhood method |
| Peru | 185 | 94-00 | Indirect sisterhood method |
| Suriname | 226 | 91-93 | CEMD |
| Uruguay | 35 | 02-02 | Cross-sectional |
| Venezuela | 67 | 01-01 | Cross-sectional |

*Associated member of WHO

| **Country** | **MMR** | **Period** | **Design** |
| --- | --- | --- | --- |
| **Northern America** | | | |
| Canada | 3 | 00-00 | Cross-sectional |
| United States of America | 9 | 02-02 | Cross-sectional |
| **Oceania** | | | |
| Australia | 4 | 98-00 | Cross-sectional |
| New Zealand | 9 | 00-00 | Cross-sectional |
